# Exome-wide analysis identifies three low-frequency missense variants associated with pancreatic cancer risk in Chinese populations

**DOI:** 10.1038/s41467-018-06136-x

**Published:** 2018-09-11

**Authors:** Jiang Chang, Jianbo Tian, Ying Zhu, Rong Zhong, Kan Zhai, Jiaoyuan Li, Juntao Ke, QiangQiang Han, Jiao Lou, Wei Chen, Beibei Zhu, Na Shen, Yi Zhang, Yajie Gong, Yang Yang, Danyi Zou, Xiating Peng, Zhi Zhang, Xuemei Zhang, Kun Huang, Ming Yang, Li Wang, Chen Wu, Dongxin Lin, Xiaoping Miao

**Affiliations:** 1 Department of Epidemiology and Biostatistics, Key Laboratory for Environment and Health, School of Public Health, Tongji Medical College, Huazhong University of Sciences and Technology, 430030 Wuhan, China; 2 Department of Etiology and Carcinogenesis, National Cancer Center/Cancer Hospital, Chinese Academy of Medical Sciences and Peking Union Medical College, 100021 Beijing, China; 3 Medical Research Center, Beijing Chao-Yang Hospital, Capital Medical University, 100020 Beijing, China; 4 Wuhan GeneCreate Biological Engineering Co., Ltd, 430075 Wuhan, China; 5 Department of Chemotherapy and Radiotherapy, Tangshan Gongren Hospital, 063210 Tangshan, China; 6 Department of Molecular Genetics, College of Life Science, North China University of Science and Technology, 063210 Tangshan, China; 7 Tongji School of Pharmacy, Huazhong University of Science and Technology, 430030 Wuhan, China; 8 Shandong Provincial Key Laboratory of Radiation Oncology, Cancer Research Center, Shandong Cancer Hospital affiliated to Shandong University, Shandong Academy of Medical Sciences, 250117 Jinan, China; 9 Department of Epidemiology and Biostatistics, Institute of Basic Medical Sciences, Chinese Academy of Medical Sciences and School of Basic Medicine, Peking Union Medical College, 100730 Beijing, China

**Keywords:** Pancreatic cancer, Genome-wide association studies

## Abstract

Germline coding variants have not been systematically investigated for pancreatic ductal adenocarcinoma (PDAC). Here we report an exome-wide investigation using the Illumina Human Exome Beadchip with 943 PDAC cases and 3908 controls in the Chinese population, followed by two independent replicate samples including 2142 cases and 4697 controls. We identify three low-frequency missense variants associated with the PDAC risk: rs34309238 in *PKN1* (OR = 1.77, 95% CI: 1.48–2.12, *P* = 5.35 × 10^−10^), rs2242241 in *DOK2* (OR = 1.85, 95% CI: 1.50–2.27, *P* = 4.34 × 10^−9^), and rs183117027 in *APOB* (OR = 2.34, 95% CI: 1.72–3.16, *P* = 4.21 × 10^−8^). Functional analyses show that the *PKN1* rs34309238 variant significantly increases the level of phosphorylated PKN1 and thus enhances PDAC cells' proliferation by phosphorylating and activating the FAK/PI3K/AKT pathway. These findings highlight the significance of coding variants in the development of PDAC and provide more insights into the prevention of this disease.

## Abstract (web summary)

Pancreatic ductal adenocarcinoma is a lethal human cancer with a poor 5-year overall survival rate. Here the authors perform an exome-wide analysis in a cohort of PDAC patients to identify three novel missense variants in *PKN1*, *DOK2*, and *APOB* genes, that are associated with PDAC risk.

## Introduction

Pancreatic ductal adenocarcinoma (PDAC) is one of the most lethal human cancers, with a 5-year overall survival rate of only ~5%^1,2^. The incidence of PDAC is rapidly increasing worldwide, and prevention or early diagnosis at a curable stage remains exceedingly difficult for this disease. Therefore, PDAC has become the fourth leading cause of cancer-associated death in both men and women^3,4^. Cigarette smoking, type 2 diabetes, obesity and several hereditary cancer syndromes represent major risk factors for PDAC^2,5–7^. Based on accumulating evidence, germline variants also play an important role in the development of this disease^8^.

In previous genome-wide association studies (GWAS) from our group and other researchers, several susceptibility loci associated with PDAC risk were identified in populations of Asian and European ancestry populations^9–15^. However, GWAS exclusively focused on common single-nucleotide polymorphisms (SNPs) with a minor allele frequency (MAF) > 5%, and the identified variants explained only a small fraction of the heritability for PDAC^16,17^. Low-frequency variants (defined here as an MAF of 0.1%–5%) or rare variants (defined here as a MAF < 0.1%) have essential effect size and may substantially contribute to the “missing” heritability^16,18^. Therefore, identifying additional low-frequency or rare variants that increase the susceptibility to PDAC will deepen our understanding of the aetiology of this disease.

The Illumina HumanExome Beadchip (referred to as “exome chip” hereafter) platform is one approach that primarily focuses on low-frequency or rare variants in the exon regions of genes, which has been successfully used in numerous studies to identify a series of functional coding variants^19–21^. In this study, we performed an exome-wide association analyses using this chip with 943 individuals with PDAC and 3908 healthy controls to identify protein-coding susceptibility loci in the Chinese population, followed by two independent replicate samples including 2142 cases and 4697 controls. We identify three low-frequency missense variants in the protein kinase N1 (*PKN1*), the docking protein 2 (*DOK2*) and the apolipoprotein B (*APOB*) associated with the PDAC risk with genome-wide significance and relatively high effect sizes (odds ratio (OR) > 1.5) by an additive model in logistic regression analysis. Further functional analyses show that the *PKN1* rs34309238 variant increases the level of phosphorylated PKN1 and thus enhances cells' proliferation by phosphorylating and activating the focal adhesion kinase (FAK)/phosphatidylinositol-3 kinase (PI3K)/AKT signalling pathway. These findings highlight the significance of low-frequency missense variants in the development of PDAC and provide more insights into the prevention of this disease.

## Results

### Three low-frequency missense SNPs were identified for PDAC

**Fig. 1:**
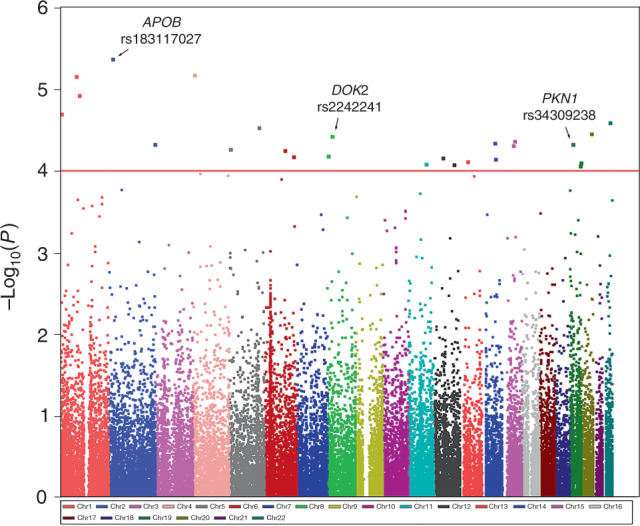
Manhattan plot for associations between genetic variants and pancreatic cancer risk. A total of 43,045 variants that passed the quality control and with MAF > 0.1% in controls were analysed and plotted. The associations (–log_10_ (*P*) values, *y* axis) are plotted against genomic position (*x* axis by chromosome and the chromosomal position of NCBI build 37). The red horizontal line corresponds to a *P* value threshold of 1.00 × 10^−4^. Variants that passed the threshold and were successfully verified in this study were annotated

In the discovery stage of this study, we performed an exome-wide association analyses in 943 individuals with PDAC and 3908 healthy controls (Supplementary Fig. 1 and Supplementary Table 1), and the cases and controls of Han Chinese ancestry were well matched (Supplementary Figs. 2, 3). The overall association *P* values are presented in Fig. 1, and 25 variants exhibited a promising association, with *P* < 1 × 10^−4^ by an additive model in logistic regression analysis (Supplementary Table 2). We therefore chose these 25 variants for further replication in 1048 cases and 2094 controls from Wuhan, and significant associations with four coding variants were verified (Supplementary Table 3). These four variants were then replicated in stage II with 1094 cases and 2603 controls from Shandong and Hebei provinces (Supplementary Table 3). These four variants were all associated with PDAC risk with same direction for both the two replication stages, and three of them with *P* < 0.05 in both the two replication stages by an additive model in logistic regression analysis. When combining the results from the discovery and replication stages, we identified three low-frequency coding variants that were significantly associated with the risk of PDAC and displayed *P* values reaching genome-wide significance by an additive model in logistic regression analysis (Table 1 and Supplementary Table 4). The most significant association was noted for rs34309238, which is located in the 11th exon of *PKN1* in chromosome 19p13.12 (OR = 1.77, 95% confidence interval (CI) 1.48–2.12, *P* = 5.35 × 10^−10^) by an additive model in logistic regression analysis. The rs2242241 variant in the fourth exon of *DOK2* and rs183117027 variant in the 28th exon of *APOB* were also associated with an increased risk of PDAC, with ORs being 1.85 (95% CI 1.50–2.27, *P* = 4.34 × 10^−9^) and 2.34 (95% CI 1.72–3.16, *P* = 4.21 × 10^−8^) by an additive model in logistic regression analysis, respectively.

**Table 1:** The identified variants associated with pancreatic cancer risk in the discovery, replication and combined samples

Chr	SNP	Gene	Allele	Variation	Stage	MAF		OR (95% CI)	*P*
						Cases	Controls		
19p13.12	rs34309238	*PKN1*	C>A	p.Leu555Ile	Discovery	0.033	0.017	1.96 (1.44–2.67)	1.79 × 10^−5^
					Replication I	0.031	0.019	1.62 (1.16–2.26)	0.0043
					Replication II	0.032	0.018	1.75 (1.28–2.40)	0.0005
					Combined	0.032	0.018	1.77 (1.48–2.12)	5.35 × 10^−10^
8p21.3	rs2242241	*DOK2*	T>G	p.Ser394Ala	Discovery	0.030	0.013	2.47 (1.75–3.49)	2.94 × 10^−7^
					Replication I	0.023	0.014	1.64 (1.13–2.40)	0.0101
					Replication II	0.022	0.014	1.51 (1.06–2.15)	0.0210
					Combined	0.025	0.014	1.85 (1.50–2.27)	4.34 × 10^−9^
2p24.1	rs183117027	*APOB*	G>A	p.Val4006Ile	Discovery	0.016	0.006	2.95 (1.83–4.74)	8.05 × 10^−6^
					Replication I	0.011	0.005	2.20 (1.21–3.98)	0.0093
					Replication II	0.011	0.006	2.01 (1.17–3.48)	0.0120
					Combined	0.012	0.006	2.34 (1.72–3.16)	4.21 × 10^−8^

*P* values are two sided and were calculated by an additive model in logistic regression analysis adjusted for sex and age

*Chr* chromosomal region, *MAF* minor allele frequency, *OR* odds ratio, *CI* confidence interval, *Allele* Reference allele > Effect allele

### No other independent signals in the significant regions

We performed an imputation analysis for the identified three regions to investigate whether the association of each of the three susceptibility regions with PDAC risk was completely explained by the index SNP. After imputation, we tested 6675 SNPs (108 directly genotyped and 6567 well-imputed SNPs) for the association with these three regions. Only two imputed variants passed our significance threshold in the discovery stage (*P* < 1 × 10^−4^ by an additive model in logistic regression analysis), and they were all in high linkage disequilibrium (LD) with the identified SNP (Supplementary Figs. 4–6 and Table 5). After conditioning with each of the three SNPs, the *P* values for the association of those SNPs in LD with the identified SNP were not <0.05, suggesting that the association signals in these regions probably point towards these three SNPs identified by genotyping (Supplementary Table 5).

### No other signals were identified by gene-based analysis

We performed a gene-based analysis to identify significant susceptible variants enriched in genes using two methods: a simple burden test and a sequence kernel association test (SKAT). A total of 24,636 variants enriched in 9647 genes were analysed. Five genes (*DOK2*, *PKN1*, *TAS1R3*, *MLPH* and *CHPT1*) passed our significance threshold in the discovery stage with *P* < 1 × 10^−4^ in either burden test or SKAT (Supplementary Table 6). However, all of the top variants of these genes were identified and replicated in the single variant analysis. We did not find novel significant susceptible variants enriched in genes.

### The rs34309238 variant may affect PKN1 phosphorylation

**Fig. 2:**
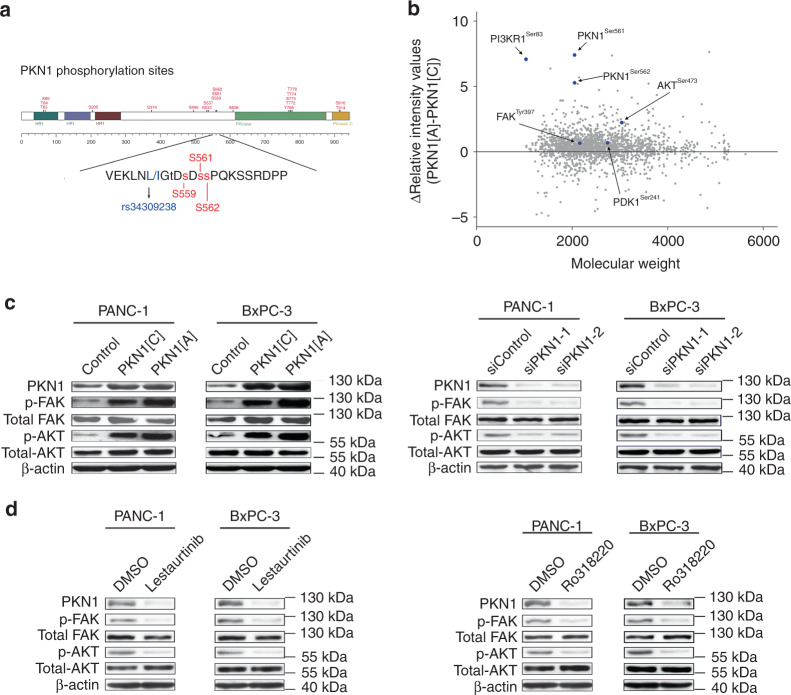
*PKN1* rs34309238 variant influences pancreatic cancer risk by altering the level of phosphorylated PKN1 and thus affecting the FAK/PI3K/AKT signalling pathway. **a** Protein modification sites of PKN1. Annotations were obtained from the PhosphoSitePlus database. **b** Result of the iTRAQ-based comparative proteomics screen. PANC-1 cells were seeded in six-well plates after transfection with PKN1[A], PKN1[C] or control vector. The raw intensity values of cells transfection with PKN1[A] or PKN1[C] were divided by the intensity values of cells transfection with control vector to obtain the relative intensity values. The *y* axis shows the relative intensity values of cells' transfection with PKN1[A] minus the relative intensity values of cells' transfection with PKN1[C]. The *x* axis shows the molecular weight of detected peptides. The proteomics screen experiment was repeated independently for two times with similar results. **c** Levels of phosphorylated FAK and AKT were affected by the *PKN1* rs34309238 variant. Cells were seeded in six-well plates after transfection with PKN1[A], PKN1[C] or control vector (left) and PKN1-targeting siRNAs or control siRNA (right). **d** Levels of phosphorylated FAK and AKT were reduced by the *PKN1* inhibitors. Cells were seeded in six-well plates after transfection with PKN1 inhibitors Lestaurtinib and Ro318220 or DMSO as control. For **c**, **d**, the western blot experiment was repeated independently for three times with similar results

Among the three identified variants, the rs34309238 variant (Leu555Ile change) in *PKN1* exhibited the most significant signal by an additive model in logistic regression analysis in this study. This variant was predicted to be probably damaging (score = 0.997 calculated by PolyPhen2). Three previously reported phosphorylation sites (S559, S561 and S562, annotated by the PhosphoSitePlus database^22^ with >5 references) are located near this variant, and thus their phosphorylation levels may be affected (Fig. 2a). Furthermore, by using data obtained from the Oncomine database and the Human Protein Atlas database, we found that PKN1 expression was upregulated in numerous cancers, such as glioma, kidney cancer, ovarian cancer, prostate cancer, and PDAC (Supplementary Fig. 7). Therefore, we conjectured that the rs34309238 C>A (Leu>Ile) change might affect PDAC risk by affecting the level of phosphorylated PKN1 and the phosphorylation of PKN1 activates its downstream signalling pathway.

### The rs34309238-A activates the PKN1/FAK/PI3K/AKT pathway

To elucidate the function of rs34309238 in the development of PDAC, we performed an isobaric Tag for Relative and Absolute Quantitation (iTRAQ)-based comparative proteomics screen for PANC-1 cells after transfection with a pcDNA3.1 plasmid containing *PKN1* rs34309238[C], rs34309238[A] or the control vector. The levels of phosphorylated PKN1 at Ser561 and Ser562 (near the rs34309238 Leu555Ile change) were increased upon transfection with PKN1[A], compared with cells transfected with PKN1[C] or the control vector (Fig. 2b). We also observed increased levels of phosphorylated FAK (FAK^Tyr397^), PI3K regulatory subunit alpha (PI3KR1^Ser83^), 3-phosphoinositide-dependent protein kinase 1 (PDK1^Ser241^) and serine/threonine kinase 1 (AKT^Ser473^) upon transfection with PKN1[A] in the iTRAQ-based screen (Fig. 2b). The phosphorylation level of FAK^Tyr397^ and AKT^Ser473^ were further successfully validated using western blot (Fig. 2c). Meanwhile, knockdown of PKN1 by small interfering RNAs (siRNAs) reduced the levels of phosphorylated FAK^Tyr397^ and AKT^Ser473^ (Fig. 2c).

### The rs34309238-A promotes PDAC cells' proliferation

**Fig. 3:**
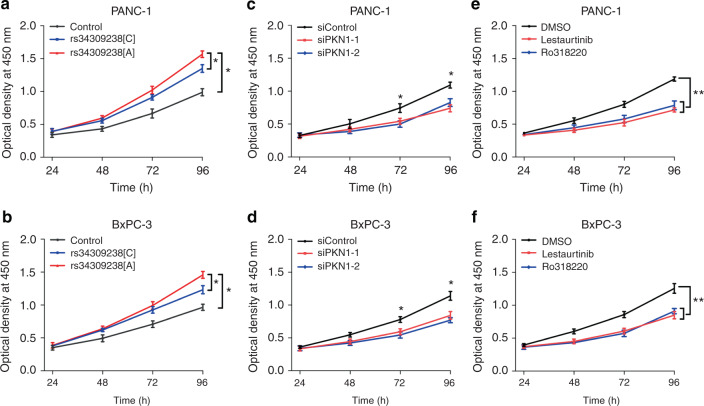
*PKN1* rs34309238 variant influences pancreatic cancer cells' proliferation. **a**, **b** Overexpression of PKN1[A] substantially enhanced the rate of cell proliferation in PANC-1 (**a**) and BxPC-3 (**b**) cells. Cells were seeded in 96-well plates after transfection with PKN1[A], PKN1[C] or control vector. **c**, **d** Knockdown of PKN1 significantly reduced the proliferation of PANC-1 (**c**) and BxPC-3 (**d**) cells. Cells were seeded in 96-well plates after transfection with PKN1-targeting siRNAs or control siRNA (siControl). **e**, **f** PKN1 inhibitors significantly reduced the rate of cell proliferation in PANC-1 (**e**) and BxPC-3 (**f**) cells. Cells were seeded in 96-well plates after transfection with PKN1 inhibitors Lestaurtinib and Ro318220 or DMSO as control. For **a**–**f**, cell numbers were determined every 24 h for 96 h using CCK-8 assays and the results present means ± s.e.m. from three independent experiments and each had six replications. *P < 0.05, **P < 0.01, compared with the control by two-sided unpaired Student’s *t* test

To further characterize the function of PKN1 variant in PDAC cells, we overexpressed different *PKN1* variants in PANC-1 and BxPC-3 cells and tested the rate of cell proliferation. The PKN1[A] overexpression significantly enhanced PANC-1 and BxPC-3 cells' proliferation compared with overexpression of PKN1[C] or the control vector by two-sided unpaired Student’s *t* test (Fig. 3a, b). In contrast, knockdown of PKN1 by two PKN1 siRNAs reduced PANC-1 and BxPC-3 cells' proliferation (Fig. 3c, d). We also selected two previously reported PKN1 inhibitors (Lestaurtinib and Ro318220)^23–25^ and tested whether they can reduce PDAC cells' proliferation. The results exhibited that both Lestaurtinib and Ro318220 inhibited PANC-1 and BxPC-3 cells' proliferation by reducing the levels of phosphorylated FAK/PI3K/AKT signalling pathway (Figs. 2d and 3e, f).

## Discussion

In this study, we used the the Illumina HumanExome Beadchip to perform an exome-wide interrogation of coding susceptibility loci for PDAC. This chip was designed based on exome sequencing data of ~12,000 individuals from the European, African, Chinese and Hispanic population. The chip consists of >240,000 markers that is estimated to include 97–98% of the nonsynonymous variants detected in average genome through exome sequencing. By using this approach, we identified three low-frequency missense variants associated with PDAC risk in a total of 3085 cases and 8605 controls. No common variants were successfully verified under the significant threshold (*P* < 0.0001 by an additive model in logistic regression analysis) in the discovery stage of this study. Potential susceptible variants with *P* values between 0.05 and 0.0001 still need to be investigated in future studies.

Among these three identified variants, the rs34309238 variant (Leu555Ile change) in *PKN1* exhibited the most significant signal. The *PKN1* located in the 19p13.12 region, which contains an important G protein-coupled receptor and cancer metastasis-related gene, CD97^26^. The PKN1 belongs to the protein kinase C superfamily and is activated upon binding a member of the Rho family of small G proteins, such as Ras-related C3 botulinum toxin substrate 1 (RAC1)^27^, which is required for KRAS-induced pancreatic tumorigenesis in mice^28^. Through a series of functional analyses, we found that the rs34309238 variant influences the risk of PDAC by altering the level of phosphorylated PKN1 at Ser561 and Ser562. The enhanced phosphorylation of PKN1 activates the FAK/PI3K/AKT signalling pathway and enhances PDAC cells' proliferation. These results are consistent with previous findings that FAK contains a phosphorylation consensus motif for PKN1^29^, and its phosphorylation enhances cancer development by activating the PI3K/AKT signalling pathway^30–33^. Our results also suggested that PKN1 inhibitors Lestaurtinib and Ro318220 reduces PDAC cells' proliferation by inhibition of PKN1-associated FAK/PI3K/AKT signalling pathway. Therefore, these two PKN1 inhibitors may serve as potential drugs for the treatment of PDAC.

In addition to the functional variant at *PKN1*, we also identified two low-frequency coding variants that were significantly associated with the risk of PDAC: rs2242241 in *DOK2* and rs183117027 in *APOB*. The *DOK2* gene is located on chromosome 8p21.3, which is frequently lost in multiple human cancers^34–36^. The DOK family members DOK1, DOK2 and DOK3 are substrates of dozens of crucial protein tyrosine kinases and function in negative-feedback signalling loops that tightly modulate the duration and intensity of growth factor signalling. *DOK2* is a tumour-suppressor gene in lung adenocarcinoma and myelomonocytic leukaemia^37,38^. Except for *DOK2*, this region contains the glial cell line-derived neurotrophic factor family receptor alpha 2, which could prompt pancreatic cancer cell growth and chemoresistance through downregulating tumour-suppressor gene PTEN via Mir-17-5p^39^.

The *APOB* gene is located in the 2p24.1 region, which contains a Barrett’s oesophagus susceptibility gene, the growth differentiation factor 7^40^. The *APOB* encodes the main apolipoprotein of chylomicrons and low-density lipoproteins. APOB exists in plasma as two main isoforms: APOB-48 and APOB-100. The former is synthesized exclusively in the gut, and the latter is synthesized exclusively in the liver. Significant APOB alterations induced by somatic mutations (~10%) or downregulation by hypermethylation likely result in hepatocellular carcinoma by diverting energy into cancer-relevant metabolic pathways^41,42^. Germline mutations in *APOB* also potentially cause diseases associated with lipid metabolic disorders, such as hypobetalipoproteinaemia and hypercholesterolaemia^43–45^. Common polymorphisms in the *APOB* gene are associated with low-density lipoprotein cholesterol metabolism or the risk of coronary heart disease^46,47^. Recently, an exome-wide associated study also identified that low-frequency coding variants in *APOB* is associated with plasma lipid level in 47,532 East Asian individuals^48^. As abnormal lipid metabolism promotes pancreatic tumorigenesis^49,50^ and obesity is an important risk factor for the PDAC^7^, rs183117027 variant may affect the development of PDAC by disturbing the lipid metabolic function of *APOB*.

Recently, an exome-wide association study based on exome and genome sequencing data identified that rare variants enriched in *BRCA2* was significantly associated with PDAC risk in the European population^51^. However, all of the identified *BRCA2* rare risk variants in this European study did not have frequency in the Chinese population. We also did not find significant *BRCA2*-susceptible variants using either single variant analysis or gene-based analysis (all *P* > 0.05). These results suggested that the rare susceptibility variants were usually population-specific^48^.

In summary, our study has identified three low-frequency coding variants that are significantly associated with PDAC susceptibility. In further functional analyses, we found for the first time that the rs34309238 variant increases the risk of PDAC by enhancing the level of phosphorylated PKN1 and thus activating the FAK/PI3K/AKT signalling pathway. These findings highlight the significance of rare coding variants in the development of PDAC and may be useful for the prevention and treatment of this disease in future.

## Methods

### Study subjects

We conducted a three-stage case–control study in the present work, and the study subjects and work flow are summarized in Supplementary Fig. 1 and Table 1. In the discovery stage, 943 patients with PDAC were recruited from Cancer Hospital, Chinese Academy of Medical Sciences in Beijing. The sample size has 52–98% power to detect variants with MAF ranging from 0.01 to 0.05 (OR = 1.8). In the replication stage I, 1048 patients with PDAC were recruited from multiple hospitals in Wuhan. The replication stage II contains 1094 patients recruited from multiple hospitals in Shandong and Hebei province. The PDAC diagnosis was confirmed histopathologically or cytologically by at least two local pathologists, according to the World Health Organization classification. A subset of individuals was included in our previous studies^11,52–55^. All the controls were cancer-free individuals selected from a community nutritional survey in the same region during the same period the patients were recruited. Demographic data were obtained from the medical records and interviews. Informed consent was obtained from all participants, and this study was approved by the institutional review board of each participating institution.

### Genotyping and quality control

In the discovery stage, samples were genotyped using the Illumina HumanExome Beadchip system to identify potential susceptibility variants. The case and control samples were mixed and randomly allocated in the plates. All initial genotyping reactions of cases and controls were performed simultaneously on the same platform, and researchers performing the assays were blinded to the case/control status. Genotype calling and quality control procedures were performed according to a standard protocol^56^.

In summary, A total of 174,391 variants were excluded from subsequent analyses because they (1) had duplicate variants on the chip (831 variants), (2) were mitochondrial variants or were located on the X or Y chromosome (1338 variants), (3) were monomorphic in our study subjects (171,141 variants), (4) had a call rate of <95% (761 variants), or (5) presented a *P* value <0.0001 in a Hardy–Weinberg equilibrium test among the control subjects (320 variants). Five PDAC cases and three controls were excluded because they (1) had an overall genotyping rate of <95% (Supplementary Fig. 1). We further excluded 30,434 variants with extremely rare MAF (<0.1%), and finally, 43,045 variants were analysed and plotted in the Manhattan plot (Supplementary Fig. 1).

Genotyping consistency in the discovery stage was assessed based on of 300 replicate samples genotyped using both exome chip and Sequenom MassAarray platform (San Diego, CA, USA) for the 25 promising variants, and the concordance rate of each variant was between 99.7% and 100%. A principal component analysis was performed using EIGENSOFT to determine ancestry and population stratification^57^ based on 4431 autosomal informative ancestry markers included in the exome chip^56^. We determined identity-by-state similarity to estimate the cryptic relatedness or duplication for each pair of samples using the PLINK software and no duplicated individuals (PI_HET > 0.25) were identified in this study.

In the first replication stage, 25 promising SNPs were genotyped in 1048 PDAC cases and 2094 controls using the Sequenom genotyping platform. In the second replication stage, 4 promising SNPs were genotyped in 1094 PDAC cases and 2603 controls using TaqMan assays platform (ABI 7900HT system, Applied Biosystems). Several genotyping quality controls were implemented in the replication stage, including (i) case and control samples were mixed in the plates, and persons who performed the genotyping assay were unaware of the case or control status; (ii) positive and negative (no DNA) samples were included on every 384-well assay plate; and (iii) direct sequencing of PCR products was employed to replicate sets of 50 randomly selected, Sequenom-genotyped and TaqMan-genotyped samples, and the concordance rate of Sequenom and TaqMan platforms for each variant was between 98.0% and 100%.

### Association analysis

We then performed an association analysis using an additive model in a logistic regression analysis with adjustments for age and sex as well as the first three principle components. A quantile–quantile (Q–Q) plot exhibited a good match between the distributions of observed *P* values and those expected by chance (inflation factor *λ* = 1.036; Supplementary Fig. 3). Variants with *P* < 0.0001 by an additive model in logistic regression analysis were considered significant and selected for further replication (Supplementary Table 2). In the replication stage, association analyses were performed using a logistic regression model adjusted for age and sex. Four variants with *P* < 0.05 in the first replication stage were further genotyped in the second replication stage (Supplementary Table 3). The association analysis was performed with PLINK (version 1.90) and R software (version 3.3.0). The Manhattan plot was generated using Haploview^58^, and Q–Q plot was generated with the R software (version 3.3.0).

### Genotype imputation

We phased the haplotypes with SHAPEIT^59^ and performed imputations with the IMPUTE2^60,61^ software to impute ungenotyped SNPs in a 1-Mb region centred on the three identified SNPs (Supplementary Figs. 4-6 and Table 4). This analysis was based on the LD and haplotypes information from the 1000 Genomes Project Phase 3 ASN samples as references. Poorly imputed variants with an information measure (Is) < 0.40 (output from IMPUTE2 info file) were excluded from subsequent analyses. Regional plots were created using LocusZoom^62^ with hg19/1000Genomes Nov 2014 ASN for the LD analysis.

### Cell lines

PANC-1 and BxPC-3 cells were obtained from the China Center for Type Culture Collection (Shanghai, China) and were cultured in Dulbecco’s Modified Eagle’s Medium (Gibco, Grand Island, NY, USA) supplemented with 10% foetal bovine serum (Gibco, Grand Island, NY, USA) and 1% antibiotics (100 U/ml penicillin and 0.1 mg/ml streptomycin) at 37 °C in a humidified atmosphere of 5% CO_2_. Both cell lines used in this study were authenticated by short tandem repeat profiling and tested for the absence of mycoplasma contamination (MycoAlert, Lonza Rockland, ME, USA).

### Construction of reporter plasmids and transfections

The full-length *PKN1* cDNA containing the rs34309238[C] allele or rs34309238[A] allele was commercially synthesized (Genewiz, Suzhou, China) and subcloned into the BamHI and XhoI sites of the pcDNA3.1(+) vector (Invitrogen, USA) to construct a vector expression of the human *PKN1* gene (Gene ID: 5585). The resulting vectors were named PKN1[C] and PKN1[A]. The siRNA oligonucleotides targeting *PKN1* and non-targeting siRNA (Supplementary Table 7) were purchased from RiboBio (Guangzhou, China). The PKN1 inhibitors Lestaurtinib (ab142107) and Ro318220 (HY-13866A) were purchased from Abcam (Cambridge, UK) and MedChem Express (NJ, USA), respectively.

PANC-1 and BxPC-3 cells were seeded in 6-well plates at a density of 1 × 10^6^ cells per well, and 3 μg of plasmids were cotransfected into cells using Lipofectamine 3000 (Invitrogen, USA). Total protein was extracted with radioimmunoprecipitation assay (RIPA) buffer (moderate strength) and supersonic decomposition. Transfection efficiency was detected by quantitative reverse transcriptase–PCR (qRT-PCR; Supplementary Fig. 8). Total RNA was extracted from cells with TRIzol (Invitrogen, USA), according to the manufacturer’s instructions. First-strand cDNAs were synthesized using the PrimeScript 1st Strand cDNA Synthesis Kit (Takara, Japan). Relative RNA expression levels were determined by qRT-PCR using the SYBR Green method on an ABI Prism 7900 sequence detection system (Applied Biosystems, USA) with Power SYBR^TM^ Green PCR Master Mix (Applied Biosystems, USA), 50 ng of cDNA templates and 0.5 μM gene-specific primers in a 10-μl reaction mixture. Reactions were performed with an initial 30-s denaturation step at 95 °C, followed by 40 cycles of 95 °C for 5 s and 60 °C for 30 s. Independent experiments were performed in triplicate. GAPDH was employed as an internal control. The sequences of all specific primers used in qPCR are upon request.

### Antibodies and western blot

The PKN1 antibody (ab195264) was purchased from Abcam (Cambridge, UK). FAK (#3285), phospho-FAK (Tyr397, #8556), Akt (#4685) and phospho-Akt (Ser473, #4060) antibodies were purchased from Cell Signaling Technology (Beverly, MA, USA). The beta-actin antibody (60008-1-lg) was purchased from Proteintech (Wuhan, China). All antibodies were validated by the company using western blots of human cell lines. The working dilution for each antibody is listed in Supplementary Table 8. The western blot experiments were repeated independently for three times with similar results. The original full western blot plots are shown in Supplementary Fig. 9.

### Phosphopeptide enrichment and labelling

PANC-1 cells were seeded in 15-cm diameter cell plates at a density of 1 × 10^8^ cells per well and transfected with pcDNA3.1 empty vector (Control), PKN1[C] and PKN1[A], respectively. After 48 h, the cells were harvested and lysed using RIPA buffer containing protease (Complete Mini, Roche, IN, USA) and phosphatase inhibitors (PhosSTOP, Roche, IN, USA). After 10-min sonication in ice water bath, the lysed samples were centrifuged at 15,000 × *g* for 30 min at 4 °C to remove cell debris. The proteins were precipitated by adding four volumes of cold acetone. The resulting protein pellets were resuspended in 8 M urea/100 mM TEAB (pH 8.0). The proteins were then reduced with 10 mM dithiothreitol at 56 °C, alkylated with 50 mM iodoacetamide in the dark and diluted 4 times with 100 mM TEAB (pH 8.0) prior to digestion overnight with trypsin (1:50 w/w). The resulting peptides were desalted with Sep-Pak C18 column (Waters, MA, USA).

iTRAQ-based quantification was used for proteomics screen. The three peptide samples were first subjected to phosphopeptides enrichment using an Immobilized Metal Affinity Chromatography method^63^. Then these samples were labelled with an eight-plex iTRAQ Kit. The labelled samples were mixed, desalted, vacuum dried and fractionated into 12 fractions using a high-pH reversed-phase chromatography method^64^ on the Ultimate 3000 HPLC system (Thermo Scientific, CA, USA). All the samples were vacuum dried and stored at −80 °C until the liquid chromatography–tandem mass spectrometry (LC-MS/MS) analysis.

### The LC-MS/MS analysis

LC-MS/MS analysis was performed on a TripleTOF 5600+ system (SCIEX, MA, USA). Each sample was first loaded onto a trap column and then chromatographed using a 90-min gradient on an analytical column (75 μm × 15 cm, Magic C18 AQ 3-µm 120-Å). The eluted peptides were sprayed into mass spectrometer and scanned. MS data were acquired in DDA mode. MS1 spectra were collected in the range 350–1250 *m*/*z* for 250 ms. The 30 most intense precursors with charge state 2–5 were selected for fragmentation, and MS2 spectra were collected in the range 50–2000 *m*/*z* for 100 ms; precursor ions were excluded from reselection for 15 s.

Raw data were processed with ProteinPilot version 4.5 (SCIEX, MA, USA). MS and MS/MS spectra were searched against SwissProt human database using Paragon algorithm^65^. The database search was performed with the following parameters: iTRAQ 8plex (peptide labelled) for Sample type according to the experiments; Iodoacetamide for Alkylation; Trypsin for Digestion; Phosphorylation emphasis for Special factors; Thorough ID for Search effort. ProteinPilot calculates a percentage of confidence that reflects the probability that the hit is a false positive. Only high-quality peptide assignments with confidence >95% were considered in this study.

### Analysis of cell proliferation

Cells were seeded in 96-well flat-bottomed plates, and each well contained 2000 cells in 100 μl of cell suspension. After a certain time in culture, cell viability was measured using CCK-8 assays (Dojindo Laboratories, Tokyo, Japan). Each experiment included six replicates and was repeated three times.

### Statistical analysis

For functional analyses, the results are presented as means ± s.e.m. Mean values from two groups were compared using Student’s *t* test. The variance similar between the groups were tested using one-way analysis of variance. All statistical analyses of the functional data were performed using the R software (3.3.0).

## Electronic supplementary material

Supplementary Information

## Data Availability

The data that support the findings of this study have been deposited in the European Genome-phenome Archive (EGA) with accession number EGAS00001003040.
