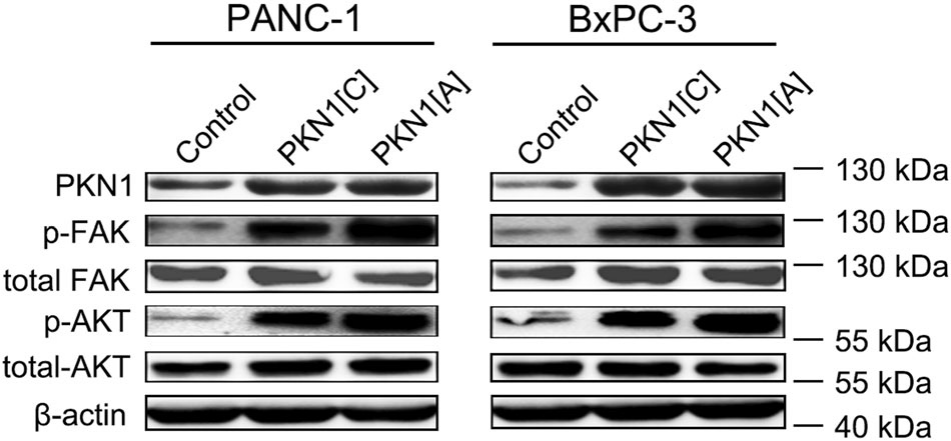# Author Correction: Exome-wide analysis identifies three low-frequency missense variants associated with pancreatic cancer risk in Chinese populations

**DOI:** 10.1038/s41467-025-58613-9

**Published:** 2025-04-11

**Authors:** Jiang Chang, Jianbo Tian, Ying Zhu, Rong Zhong, Kan Zhai, Jiaoyuan Li, Juntao Ke, QiangQiang Han, Jiao Lou, Wei Chen, Beibei Zhu, Na Shen, Yi Zhang, Yajie Gong, Yang Yang, Danyi Zou, Xiating Peng, Zhi Zhang, Xuemei Zhang, Kun Huang, Ming Yang, Li Wang, Chen Wu, Dongxin Lin, Xiaoping Miao

**Affiliations:** 1 Department of Epidemiology and Biostatistics, Key Laboratory for Environment and Health, School of Public Health, Tongji Medical College, Huazhong University of Sciences and Technology, 430030 Wuhan, China; 2 Department of Etiology and Carcinogenesis, National Cancer Center/Cancer Hospital, Chinese Academy of Medical Sciences and Peking Union Medical College, 100021 Beijing, China; 3 Medical Research Center, Beijing Chao-Yang Hospital, Capital Medical University, 100020 Beijing, China; 4 Wuhan GeneCreate Biological Engineering Co., Ltd, 430075 Wuhan, China; 5 Department of Chemotherapy and Radiotherapy, Tangshan Gongren Hospital, 063210 Tangshan, China; 6 Department of Molecular Genetics, College of Life Science, North China University of Science and Technology, 063210 Tangshan, China; 7 Tongji School of Pharmacy, Huazhong University of Science and Technology, 430030 Wuhan, China; 8 Shandong Provincial Key Laboratory of Radiation Oncology, Cancer Research Center, Shandong Cancer Hospital affiliated to Shandong University, Shandong Academy of Medical Sciences, 250117 Jinan, China; 9 Department of Epidemiology and Biostatistics, Institute of Basic Medical Sciences, Chinese Academy of Medical Sciences and School of Basic Medicine, Peking Union Medical College, 100730 Beijing, China

Correction to: *Nature Communications* 10.1038/s41467-018-06136-x, published online 11 September 2018

In the version of this article initially published, incorrect p-FAK and β-actin results for the BxPC-3 cell line were inadvertently cropped into the main text figure (Fig. 2c) from the original western blot data. Notably, the original western plots were already included in Supplementary Fig. 9 at the time of publication. To rectify this, we have now re-cropped the corrected image from Supplementary Fig. 9 and provide it below as the updated Fig. 2c.

Fig. 2c Corrected.