# Supplementary material for: Exome-wide analysis identifies three low-frequency missense variants associated with pancreatic cancer risk in Chinese populations
Source: Nat Commun. 2018 Sep 11;9:3688. doi: 10.1038/s41467-018-06136-x (PMC6134090; doi:10.1038/s41467-018-06136-x)
Supplement: Supplementary file 1 — Supplementary Information [file 41467_2018_6136_MOESM1_ESM.pdf]

Exome-wide analysis identifies three low-frequency missense variants associated with pancreatic cancer risk in Chinese populations

Chang et al.

Supplementary Table 1. Selected characteristics of cases with pancreatic cancer and controls in this study

	Discovery				Replication I				Replication II				Combined Samples			
	Cases (n = 943)		Controls (n = 3,908)		Cases (n = 1,048)		Controls (n = 2,094)		Cases (n = 1,094)		Controls (n = 2,603)		Cases (n =3,085)		Controls (n = 8,605)	
Age, mean (SD)	60.2	(11.6)	63.7	(10.2)	58.9	(12.4)	61.7	(12.2)	59.7	11.0	62.1	10.5	59.6	11.7	62.7	10.9
Gender																
Male	748	(79.3)	2,455	(62.8)	646	(61.6)	1,307	(62.4)	684	(62.5)	1,876	(72.1)	2,078	(67.4)	5,638	(65.5)
Female	195	(20.7)	1,453	(37.2)	402	(38.4)	787	(37.6)	410	(37.5)	727	(27.9)	1,007	(32.6)	2,967	(34.5)
Stage																
Local disease	116	(19.9)			98	(17.4)			113	(23.4)			327	(20.1)		
Local advanced disease	268	(46.0)			246	(43.8)			206	(42.7)			720	(44.3)		
Metastatic disease	199	(34.1)			218	(38.8)			163	(33.8)			580	(35.6)		
Unknown	360				486				612				1,458			

Supplementary Table 2. The results of variants that were found to be potentially associated with risk of pancreatic cancer at the discovery stage and were selected for further replication

Chr	SNP	Gene	Location	Major Allele	Minor Allele	MAF		OR (95% CI)†	P†	OR (95% CI)‡	P‡
						Cases	Controls				
1	rs147441599	TAS1R3	missense	C	A	0.050	0.030	1.75 (1.36−2.27)	1.95×10^{-5}	1.78 (1.38−2.29)	7.32×10^{-6}
1	rs34535037	ZZZ3	missense	A	G	0.065	0.039	1.68 (1.34−2.11)	6.73×10^{-6}	1.69 (1.35−2.10)	3.81×10^{-6}
1	rs1800549	ABCA4	missense	C	T	0.040	0.067	0.56 (0.44−0.73)	1.17×10^{-5}	0.57 (0.44−0.73)	1.05×10^{-5}
2	rs183117027	APOB	missense	G	A	0.016	0.006	3.15 (1.93−5.13)	4.16×10^{-6}	2.95 (1.83−4.74)	8.05×10^{-6}
2	rs80292002	MLPH	missense	A	G	0.015	0.031	0.43 (0.29−0.65)	4.61×10^{-5}	0.46 (0.31−0.68)	1.10×10^{-4}
4	rs2279252	EVC	missense	G	A	0.064	0.040	1.68 (1.34−2.10)	6.50×10^{-6}	1.62 (1.30−2.02)	1.63×10^{-5}
5	rs402710	CLPTM1L	intron	C	T	0.374	0.323	1.25 (1.12−1.40)	5.33×10^{-5}	1.26 (1.13−1.40)	2.57×10^{-5}
5	rs4958532	NMUR2	missense	C	T	0.061	0.038	1.64 (1.30−2.07)	2.87×10^{-5}	1.69 (1.35−2.13)	4.99×10^{-6}
6	rs1159148	AIM1	missense	A	C	0.081	0.054	1.52 (1.24−1.85)	5.53×10^{-5}	1.60 (1.31−1.95)	3.18×10^{-6}
6	rs140135976	SYNE1	missense	C	T	0.032	0.016	1.94 (1.40−2.68)	6.55×10^{-5}	2.07 (1.51−2.84)	6.01×10^{-6}
8	rs583087	CSMD1	3'−UTR	C	T	0.085	0.059	1.49 (1.22−1.81)	6.43×10^{-5}	1.48 (1.22−1.79)	5.72×10^{-5}
8	rs2242241	DOK2	missense	T	G	0.030	0.013	2.10 (1.48−3.00)	3.66×10^{-5}	2.47 (1.75−3.49)	2.94×10^{-7}
11	rs80293525	FAT3	missense	G	A	0.019	0.036	0.48 (0.34−0.69)	8.01×10^{-5}	0.49 (0.34−0.70)	1.06×10^{-4}
12	rs12829856	TWF1	synonymous	C	T	0.042	0.068	0.60 (0.47−0.77)	6.75×10^{-5}	0.60 (0.47−0.76)	3.33×10^{-5}
12	rs79716906	CHPT1	missense	T	C	0.100	0.069	1.44 (1.20−1.73)	8.19×10^{-5}	1.48 (1.24−1.77)	1.19×10^{-5}
13	rs9547952	POSTN	missense	G	A	0.061	0.088	0.66 (0.53−0.81)	7.58×10^{-5}	0.66 (0.54−0.81)	6.82×10^{-5}
14	rs11628107	SYNE2	missense	A	G	0.062	0.038	1.62 (1.28−2.04)	4.49×10^{-5}	1.65 (1.32−2.07)	1.11×10^{-5}
14	rs1465788	ZFP36L1	Upstream	G	A	0.312	0.256	1.26 (1.13−1.42)	7.01×10^{-5}	1.36 (1.22−1.53)	6.36×10^{-8}
15	rs151252589	PIGB	missense	T	G	0.017	0.041	0.46 (0.32−0.67)	4.78×10^{-5}	0.39 (0.27−0.56)	5.21×10^{-7}
15	rs11629598	VPS13C	missense	A	G	0.034	0.055	0.56 (0.42−0.74)	4.26×10^{-5}	0.58 (0.44−0.76)	8.87×10^{-5}
19	rs34309238	PKN1	missense	C	A	0.033	0.017	1.93 (1.41−2.64)	4.65×10^{-5}	1.96 (1.44−2.67)	1.79×10^{-5}
19	rs143490927	ZNF175	frameshift	-	G	0.041	0.027	1.76 (1.33−2.34)	8.53×10^{-5}	1.63 (1.23−2.14)	5.54×10^{-4}
19	rs12975366	LILRB5	missense	A	G	0.022	0.046	0.51 (0.36−0.71)	7.84×10^{-5}	0.45 (0.32−0.62)	1.97×10^{-6}
20	rs35575210	BCAS1	missense	A	C	0.143	0.109	1.39 (1.19−1.62)	3.44×10^{-5}	1.37 (1.18−1.60)	3.83×10^{-5}
22	rs55989856	APOBEC3H	5'−UTR	T	C	0.117	0.084	1.44 (1.22−1.71)	2.52×10^{-5}	1.45 (1.23−1.72)	1.03×10^{-5}

Note: Chr, chromosome; MAF, minor allele frequency; OR, odds ratio; CI, confidence interval. †*P* values are two sided and were calculated by an additive model in logistic regression analysis adjusted for sex, age and the first three principle components. ‡*P* values are two sided and were calculated by an additive model in logistic regression analysis adjusted for sex and age.

Supplementary Table 3. The results of variants that were found to be potentially associated with risk of pancreatic cancer at the replication stage

SNP	Replication I				Replication II				Combined replication samples			
	MAF		OR (95% CI)	*P*	MAF		OR (95% CI)	*P*	MAF		OR (95% CI)	*P*
	Cases	Controls			Cases	Controls			Cases	Controls		
rs147441599	0.032	0.032	0.97 (0.72−1.31)	0.8525	–	–	–	–	–	–	–	–
rs34535037	0.041	0.035	1.11 (0.85−1.45)	0.4529	–	–	–	–	–	–	–	–
rs1800549	0.054	0.063	0.85 (0.68−1.07)	0.1737	–	–	–	–	–	–	–	–
rs183117027	0.011	0.005	2.20 (1.21−3.98)	0.0093	0.011	0.006	2.01 (1.17−3.48)	0.0120	0.011	0.006	2.09 (1.40−3.12)	0.0003
rs80292002	0.018	0.028	0.64 (0.44−0.93)	0.0187	0.027	0.030	0.87 (0.64−1.18)	0.3803	0.022	0.029	0.77 (0.61−0.97)	0.0279
rs2279252	0.045	0.038	1.21 (0.93−1.57)	0.1542	–	–	–	–	–	–	–	–
rs402710	0.320	0.322	0.99 (0.88−1.11)	0.8633	–	–	–	–	–	–	–	–
rs4958532	0.033	0.042	0.80 (0.60−1.07)	0.1265	–	–	–	–	–	–	–	–
rs1159148	0.058	0.052	1.14 (0.90−1.43)	0.2771	–	–	–	–	–	–	–	–
rs140135976	0.017	0.020	0.89 (0.60−1.32)	0.5549	–	–	–	–	–	–	–	–
rs583087	0.073	0.067	1.09 (0.89−1.34)	0.3906	–	–	–	–	–	–	–	–
rs2242241	0.023	0.014	1.64 (1.13−2.40)	0.0101	0.022	0.014	1.51 (1.06−2.15)	0.0210	0.023	0.014	1.55 (1.20−2.01)	0.0007
rs80293525	0.027	0.032	0.87 (0.63−1.20)	0.3917	–	–	–	–	–	–	–	–
rs12829856	0.067	0.064	1.05 (0.85−1.29)	0.6837	–	–	–	–	–	–	–	–
rs79716906	0.057	0.066	0.85 (0.68−1.06)	0.1528	–	–	–	–	–	–	–	–
rs9547952	0.082	0.084	0.97 (0.80−1.17)	0.7475	–	–	–	–	–	–	–	–
rs11628107	0.038	0.042	0.92 (0.70−1.20)	0.5376	–	–	–	–	–	–	–	–
rs1465788	0.251	0.262	0.94 (0.83−1.06)	0.3000	–	–	–	–	–	–	–	–
rs151252589	0.040	0.037	1.09 (0.83−1.44)	0.5246	–	–	–	–	–	–	–	–
rs11629598	0.048	0.052	0.94 (0.74−1.20)	0.6382	–	–	–	–	–	–	–	–
rs34309238	0.031	0.019	1.62 (1.16−2.26)	0.0043	0.032	0.018	1.75 (1.28−2.40)	0.0005	0.031	0.018	1.70 (1.35−2.13)	5.53×10^{-6}
rs143490927	0.025	0.030	0.85 (0.61−1.18)	0.3236	–	–	–	–	–	–	–	–
rs12975366	0.048	0.046	1.08 (0.84−1.39)	0.5486	–	–	–	–	–	–	–	–
rs35575210	0.117	0.118	0.99 (0.83−1.17)	0.8777	–	–	–	–	–	–	–	–
rs55989856	0.094	0.090	1.05 (0.88−1.26)	0.5655	–	–	–	–	–	–	–	–

Note: Chr, chromosome; MAF, minor allele frequency; OR, odds ratio; CI, confidence interval. *P* values are two sided and were calculated by an additive model in logistic regression analysis adjusted for sex and age. Four SNPs with $P < 0.05$ in replication I stage were selected for further replication II stage. Three SNPs with $P < 0.05$ in both two replication stages were identified to be significantly associated with risk of pancreatic cancer in this study.

Supplementary Table 4. The identified variants associated with pancreatic cancer risk in discovery, replication and combined samples

SNP	Genotype	Discovery stage				Replication I				Replication II				Combined samples			
		Cases No. (%)	Controls No. (%)	OR (95% CI)	*P*	Cases No. (%)	Controls No. (%)	OR (95% CI)	*P*	Cases No. (%)	Controls No. (%)	OR (95% CI)	*P*	Cases No. (%)	Controls No. (%)	OR (95% CI)	*P*
rs183117027	GG	913 (96.8)	3,861 (98.8)	1.00 (Reference)		1,025 (97.8)	2,072 (98.9)	1.00 (Reference)		1,070 (97.8)	2,573 (98.8)	1.00 (Reference)		3,008 (97.5)	8,506 (98.8)	1.00 (Reference)	
	GA	30 (3.2)	47 (1.2)	2.95 (1.83−4.74)	8.05×10^{-6}	23 (2.2)	22 (1.1)	2.20 (1.21−3.98)	0.0093	24 (2.2)	30 (1.2)	2.01 (1.17−3.48)	0.0120	77 (2.5)	99 (1.2)	2.34 (1.72−3.16)	4.21×10^{-8}
	AA	0 (0.0)	0 (0.0)	–	–	0 (0.0)	0 (0.0)	–	–	0 (0.0)	0 (0.0)	–	–	0 (0.0)	0 (0.0)	–	–
rs2242241	TT	883 (94.0)	3,704 (97.4)	1.00 (Reference)		1,001 (95.5)	2,035 (97.2)	1.00 (Reference)		1,050 (96.0)	2,530 (97.2)	1.00 (Reference)		2,934 (95.2)	8,269 (97.3)	1.00 (Reference)	
	TG	56 (6.0)	97 (2.6)	2.47 (1.75−3.49)	2.94×10^{-7}	45 (4.3)	58 (2.8)	1.61 (1.08−2.40)	0.0019	39 (3.6)	71 (2.7)	1.30 (0.87−1.94)	0.1990	140 (4.5)	226 (2.7)	1.78 (1.43−2.21)	1.96×10^{-7}
	GG	0 (0.0)	0 (0.0)	–	–	2 (0.2)	1 (0.0)	3.83 (0.34−43.10)	0.2763	5 (0.5)	2 (0.1)	7.04 (1.35−36.65)	0.0205	7 (0.2)	3 (0.0)	6.73 (1.72−26.28)	0.0061
	TG+GG	–	–	–	–	47 (4.5)	59 (2.8)	1.65 (1.11−2.44)	0.0127	44 (4.0)	73 (2.8)	1.44 (0.98−2.12)	0.0616	147 (4.8)	229 (2.7)	1.84 (1.49-1.28)	1.90×10^{-8}
rs34309238	CC	883 (93.6)	3,776 (96.6)	1.00 (Reference)		987 (94.2)	2,016 (96.3)	1.00 (Reference)		1,028 (94.0)	2,512 (96.5)	1.00 (Reference)		2,898 (93.9)	8,304 (96.5)	1.00 (Reference)	
	CA	58 (6.2)	129 (3.3)	2.00 (1.44−2.77)	3.24×10^{-5}	58 (5.5)	77 (3.7)	1.54 (1.08−2.19)	0.0160	62 (5.7)	90 (3.5)	1.66 (1.19−2.33)	0.0029	178 (5.8)	296 (3.4)	1.72 (1.42−2.09)	3.10×10^{-8}
	AA	2 (0.2)	3 (0.1)	2.95 (0.48−18.20)	0.2436	3 (0.3)	1 (0.0)	6.54 (0.68−63.25)	0.1046	4 (0.4)	1 (0.0)	7.66 (0.84−69.69)	0.0707	9 (0.3)	5 (0.1)	5.02 (1.67−15.11)	0.0041
	CA+AA	60 (6.4)	132 (3.4)	2.02 (1.46−2.78)	1.85×10^{-5}	61 (5.8)	78 (3.7)	1.60 (1.13−2.26)	0.0075	66 (6.4)	91 (3.5)	1.74 (1.25−2.42)	0.0010	187 (6.1)	301 (3.5)	1.78 (1.47−2.15)	2.45×10^{-9}

Note: OR, odds ratio; CI, confidence interval. *P* values are two sided and were calculated in logistic regression analysis adjusted for sex and age.

Supplementary Table 5. Association results of variants in median LD (r^2 > 0.3) with genotype-identified SNPs in the imputation samples

Chr	SNP	Position	Gene	Location	Type	Imputation quality	MAF		OR (95% CI)	$P^{\dagger}$	$P^{\ddagger}$	r^2
							Cases	Controls				
2	rs563511124	21099983	–	intergenic	Imputation	0.77	0.006	0.002	3.41 (1.59−7.32)	0.0016	0.9329	0.50
2	rs141475848	21129509	–	intergenic	Imputation	0.71	0.003	0.001	2.49 (0.88−7.09)	0.0869	0.4155	0.30
2	rs536358437	21193624	–	intergenic	Imputation	0.92	0.016	0.006	3.15 (1.93−5.13)	4.16×10^{-6}	–	1.00
**2**	**rs183117027**	**21227212**	**APOB**	**Missense**	**Genotype**	**–**	**0.016**	**0.006**	**3.15 (1.93−5.13)**	**4.16×10^{-6}**	**–**	**1.00**
2	rs185784234	21257214	APOB	Intron	Imputation	0.88	0.010	0.003	3.95 (2.11−7.40)	1.83×10^{-5}	0.9146	0.80
8	rs75957949	21729376	–	intergenic	Imputation	0.64	0.024	0.014	1.84 (1.18−2.87)	0.0071	0.6103	0.67
8	rs190160249	21756962	–	intergenic	Imputation	0.63	0.009	0.005	1.73 (0.95−3.16)	0.0743	0.1242	0.40
**8**	**rs2242241**	**21766881**	**DOK2**	**Missense**	**Genotype**	**–**	**0.030**	**0.013**	**2.10 (1.48−3.00)**	**3.66×10^{-5}**	**–**	**1.00**
8	rs140785399	21847003	XPO7	Intron	Imputation	0.81	0.013	0.005	2.52 (1.32−4.83)	0.0052	0.8394	0.81
19	rs147893590	14547445	PKN1	Intron	Imputation	0.80	0.007	0.004	1.99 (1.00−3.96)	0.0514	0.6389	0.37
**19**	**rs34309238**	**14574897**	**PKN1**	**Missense**	**Genotype**	**–**	**0.033**	**0.017**	**1.93 (1.41−2.64)**	**4.65×10^{-5}**	**–**	**1.00**
19	rs142502013	14598070	GIPC1	Intron	Imputation	0.90	0.017	0.010	2.00 (1.29−3.12)	0.0022	0.3223	0.61

Note: These SNPs were imputed using SHAPEIT and IMPUTE2 software with LD and haplotype information based on the 1000 Genome Project Phase 3 ASN population as the reference. The imputation quality scores are obtained from the info file of the output of IMPUTE2 software. Chr, chromosome; MAF, minor allele frequency; OR, odds ratio; CI, confidence interval. $^{\dagger}P$ values are two-sided and were calculated by an additive model in logistic regression analysis with adjustment for sex and age and the first three principle components. $^{\ddagger}P$ value for conditional analysis on index SNP in bold. The LD r^2 were calculated between each variant and index SNP in bold.

Supplementary Table 6. Association results of genes that were found to be potentially associated with risk of pancreatic cancer at the discovery stage through gene-based analysis

Gene	SNPs	Single variant *P*	Burden test *P*	SKAT *P*	SKAT-O *P*
DOK2	rs2242241	$3.66×10^{-5}$	$2.38×10^{-5}$	$2.38×10^{-5}$	$2.38×10^{-5}$
PKN1	rs34309238	$4.65×10^{-5}$	$3.34×10^{-4}$	$3.24×10^{-5}$	$3.58×10^{-5}$
	rs200283627	0.1198			
	rs2230539	0.1489			
	rs200208099	0.6996			
	rs10846	0.2618			
	rs200283627	0.1198			
TAS1R3	rs147441599	$1.95×10^{-5}$	$1.40×10^{-3}$	$3.84×10^{-5}$	$7.10×10^{-5}$
	rs140037074	0.4905			
	rs200580453	0.8511			
	rs307377	0.4413			
MLPH	rs80292002	$4.61×10^{-5}$	$6.42×10^{-4}$	$6.80×10^{-5}$	$8.96×10^{-5}$
	rs2292880	0.0046			
	rs3751109	0.4027			
	rs3751108	0.6943			
	rs3751107	0.3749			
	rs200034833	0.6238			
	rs11883500	0.1202			
	rs2292884	0.3585			
	rs3817362	0.5519			
	rs61737681	0.0201			
CHPT1	rs79716906	$8.19×10^{-5}$	$7.47×10^{-5}$	$7.48×10^{-5}$	$7.54×10^{-5}$
	rs3205421	0.6357			

Note: *P* values are adjusted for sex, age and the first three principle components.

Supplementary Table 7. The sequences of siRNAs used in this study

siRNA	Target sequence (5’-3’)
PKN1-siRNA1	CCATTGAGCTGAAGGTGAA
PKN1-siRNA2	CGAGAGAGATGCAGAAGAT
PKN1-siRNA3	GTGACATATCGGTGGAGAA

Supplementary Table 8. The antibodies used in this study

Antibody	Company	Catalogue number	Working dilution
PKN1	Abcam	ab195264	1:1000
FAK	Cell Signaling Technology	3285	1:1000
phospho-FAK	Cell Signaling Technology	8556	1:1000
Akt	Cell Signaling Technology	4685	1:1000
phospho-Akt	Cell Signaling Technology	4060	1:1000
beta-actin	Proteintech	60008-1-lg	1:1000

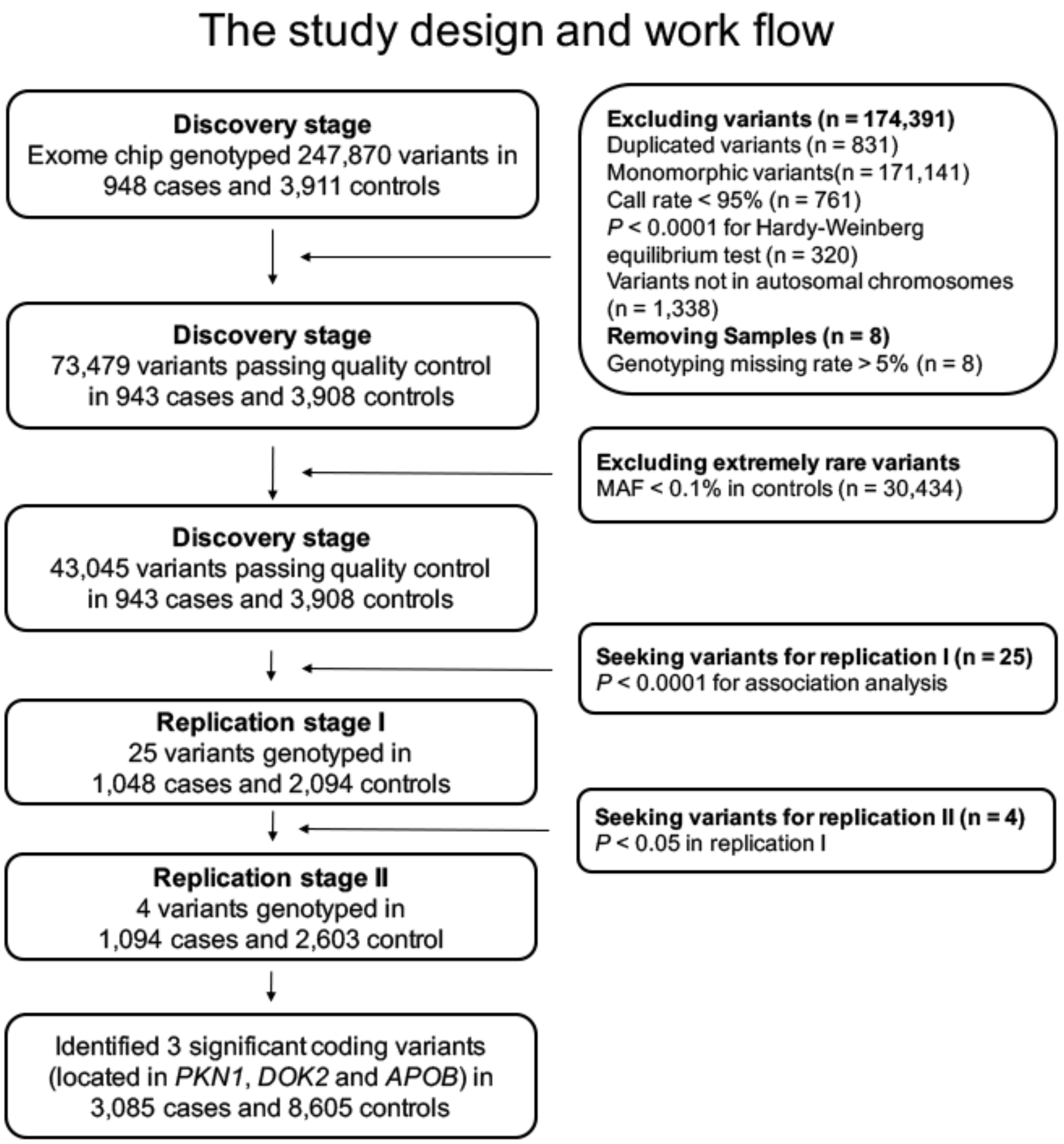

Supplementary Figure 1. Summary of the study design and work flow

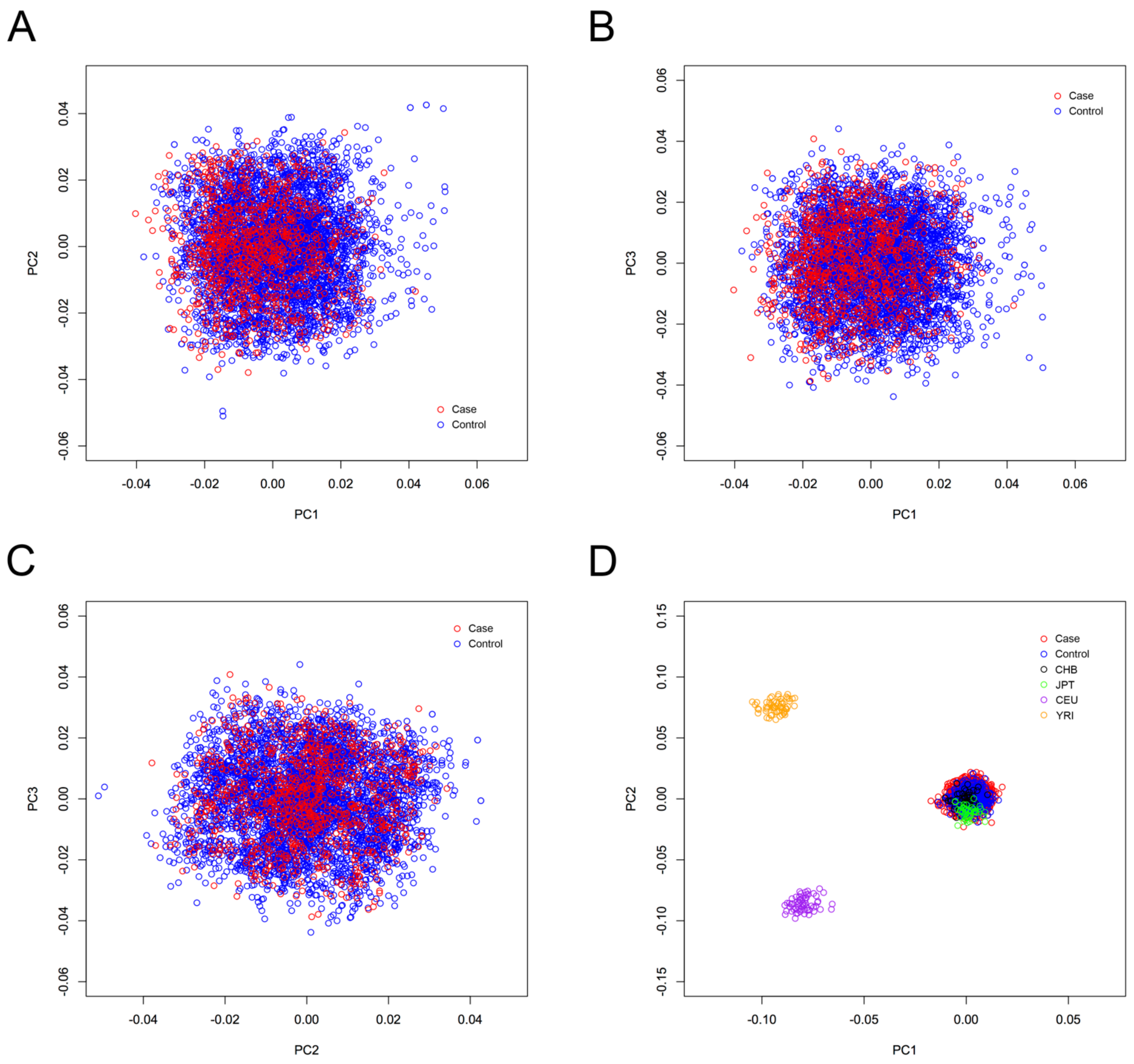

Supplementary Figure 2. Plots for genetic matching of three principal components derived from the PCA of 943 individuals with pancreatic cancer and 3,908 controls, and 206 HapMap individuals without relationships. **(A)** PC1 versus PC2 for cases and controls. **(B)** PC1 versus PC3 for cases and controls. **(C)** PC2 versus PC3 for cases and controls. **(D)** PC1 versus PC2 for cases, controls and HapMap individuals including 57 YRIs, 60 CEUs, 44 JPTs, and 45 CHBs. The case-control matching suggested minimal evidence of population stratification.

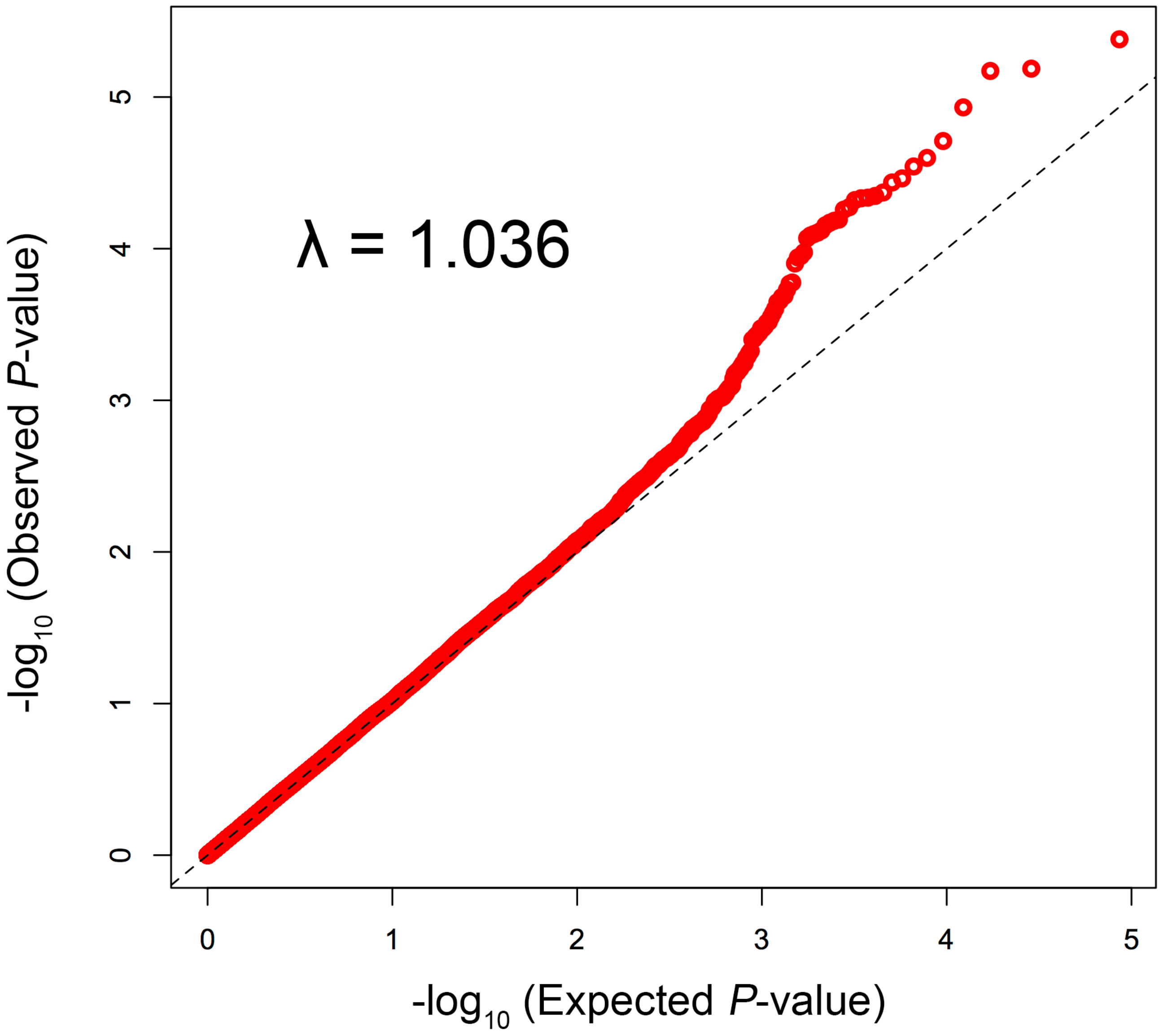

Supplementary Figure 3. Quantile-quantile plot and genomic inflation factor lambda for associations with pancreatic cancer risk. The red circles represent the distribution of *P* values for the association in the discovery stage. The observed versus expected χ^2 test statistics showed no evidence for inflation of χ^2 tests (inflation factor λ = 1.036).

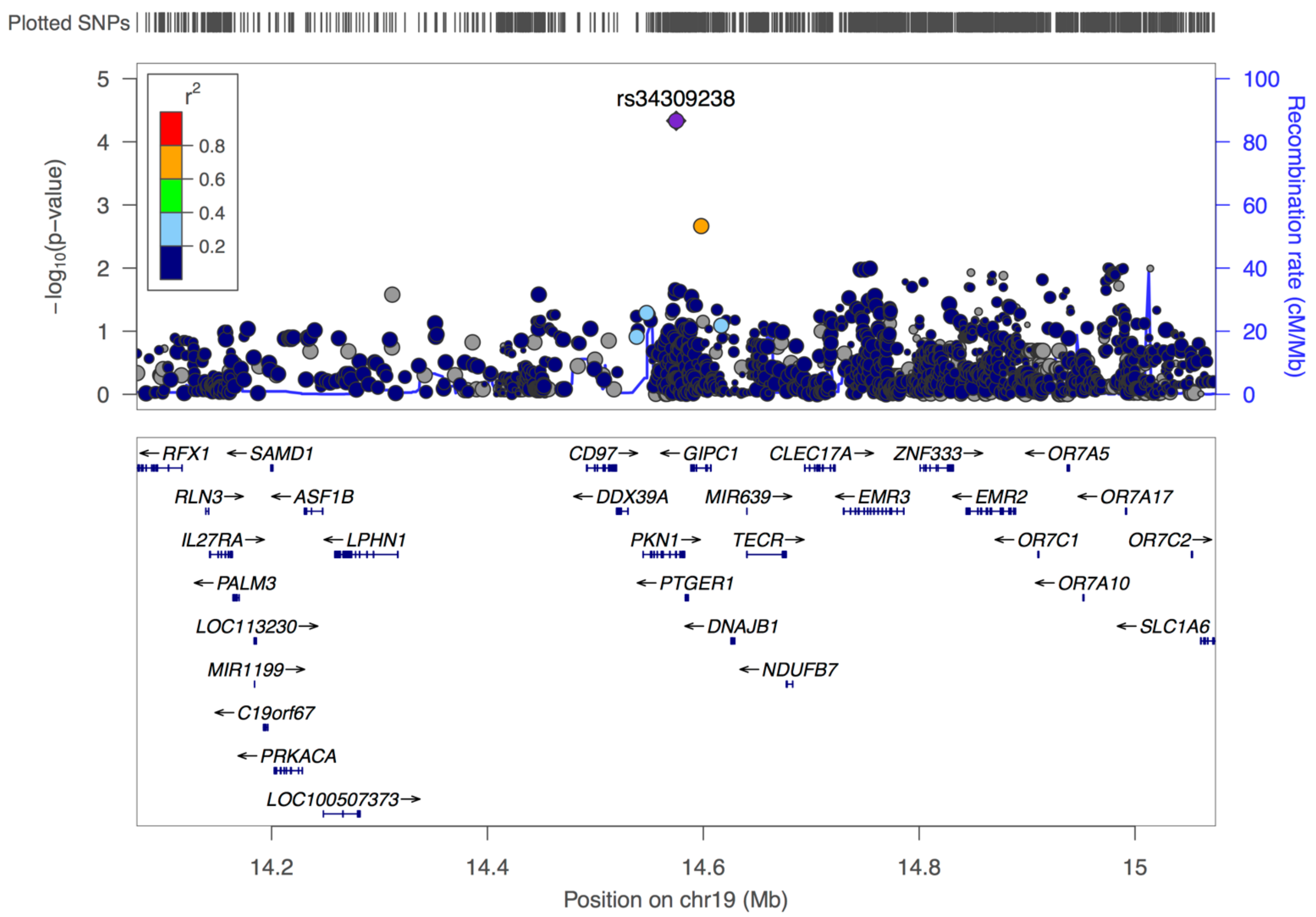

Supplementary Figure 4. Regional plot of association results and recombination rates within rs34309238. The $-\log_{10}$ *P* values (*y* axis) of the SNPs are presented according to their chromosomal positions (*x* axis). The genetic recombination rates (cM/Mb) estimated using the 1000 Genomes June 2014 ASN samples are shown with a blue line; we annotated the genes within the interested region and these genes are shown as arrows. The top genotyped SNP is labeled by rs ID and the r^2 values of the rest of the SNPs with the top genotyped SNP are indicated by different colors.

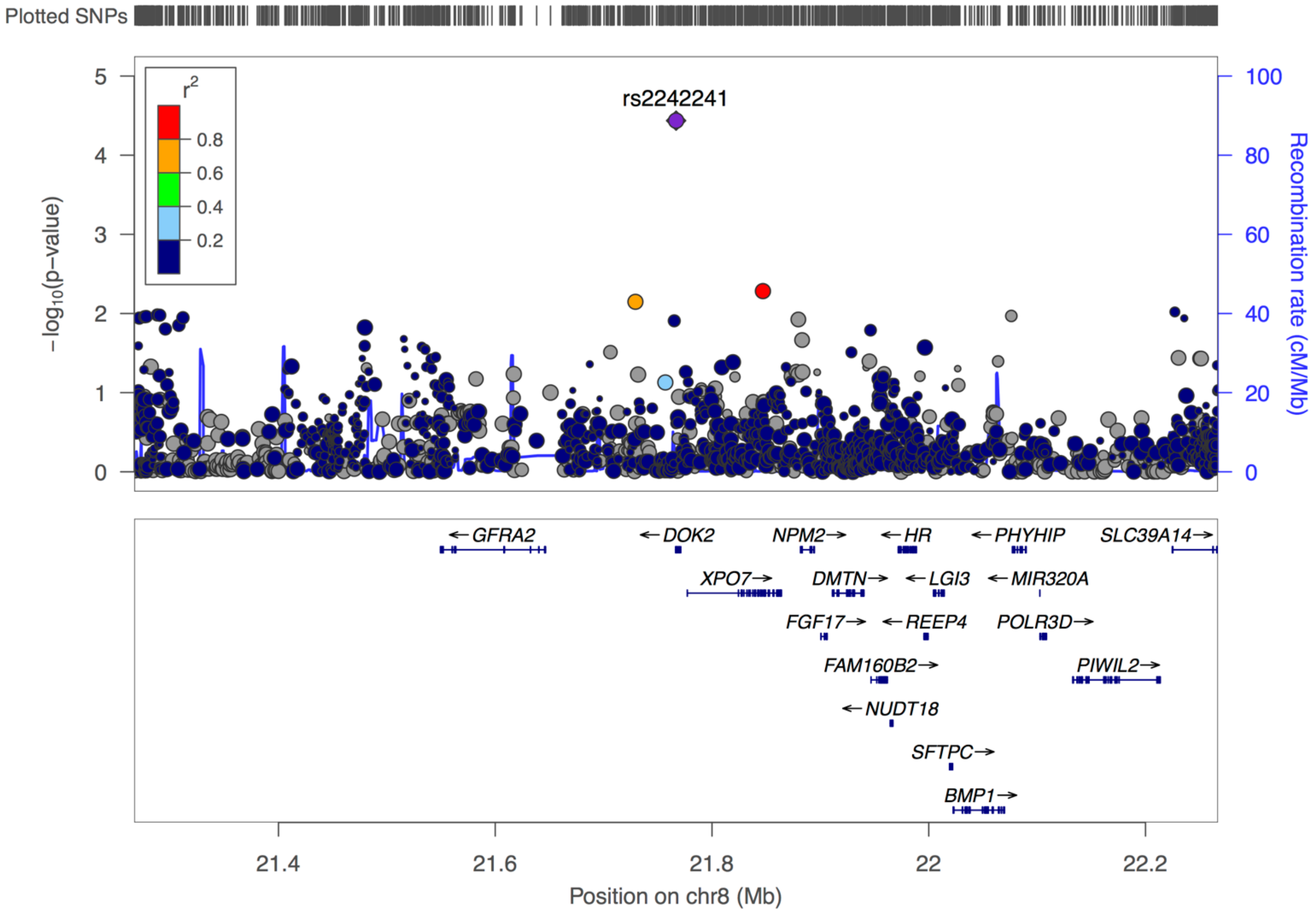

Supplementary Figure 5. Regional plot of association results and recombination rates within rs2242241. The $-\log_{10} P$ values (y axis) of the SNPs are presented according to their chromosomal positions (x axis). The genetic recombination rates (cM/Mb) estimated using the 1000 Genomes June 2014 ASN samples are shown with a blue line; we annotated the genes within the interested region and these genes are shown as arrows. The top genotyped SNP is labeled by rs ID and the r^2 values of the rest of the SNPs with the top genotyped SNP are indicated by different colors.

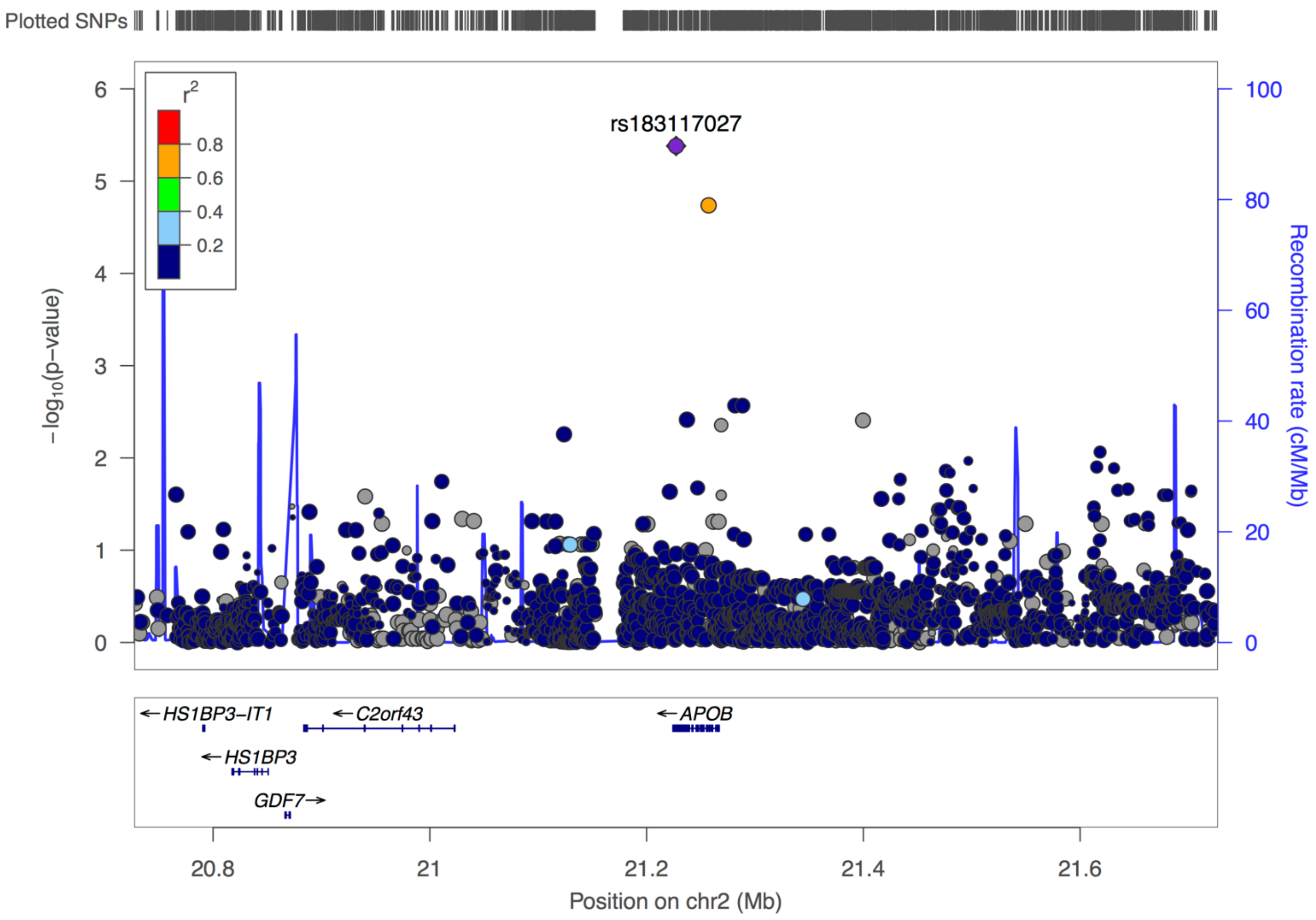

Supplementary Figure 6. Regional plot of association results and recombination rates within rs183117027. The $-\log_{10}$ *P* values (*y* axis) of the SNPs are presented according to their chromosomal positions (*x* axis). The genetic recombination rates (cM/Mb) estimated using the 1000 Genomes June 2014 ASN samples are shown with a blue line; we annotated the genes within the interested region and these genes are shown as arrows. The top genotyped SNP is labeled by rs ID and the r^2 values of the rest of the SNPs with the top genotyped SNP are indicated by different colors.

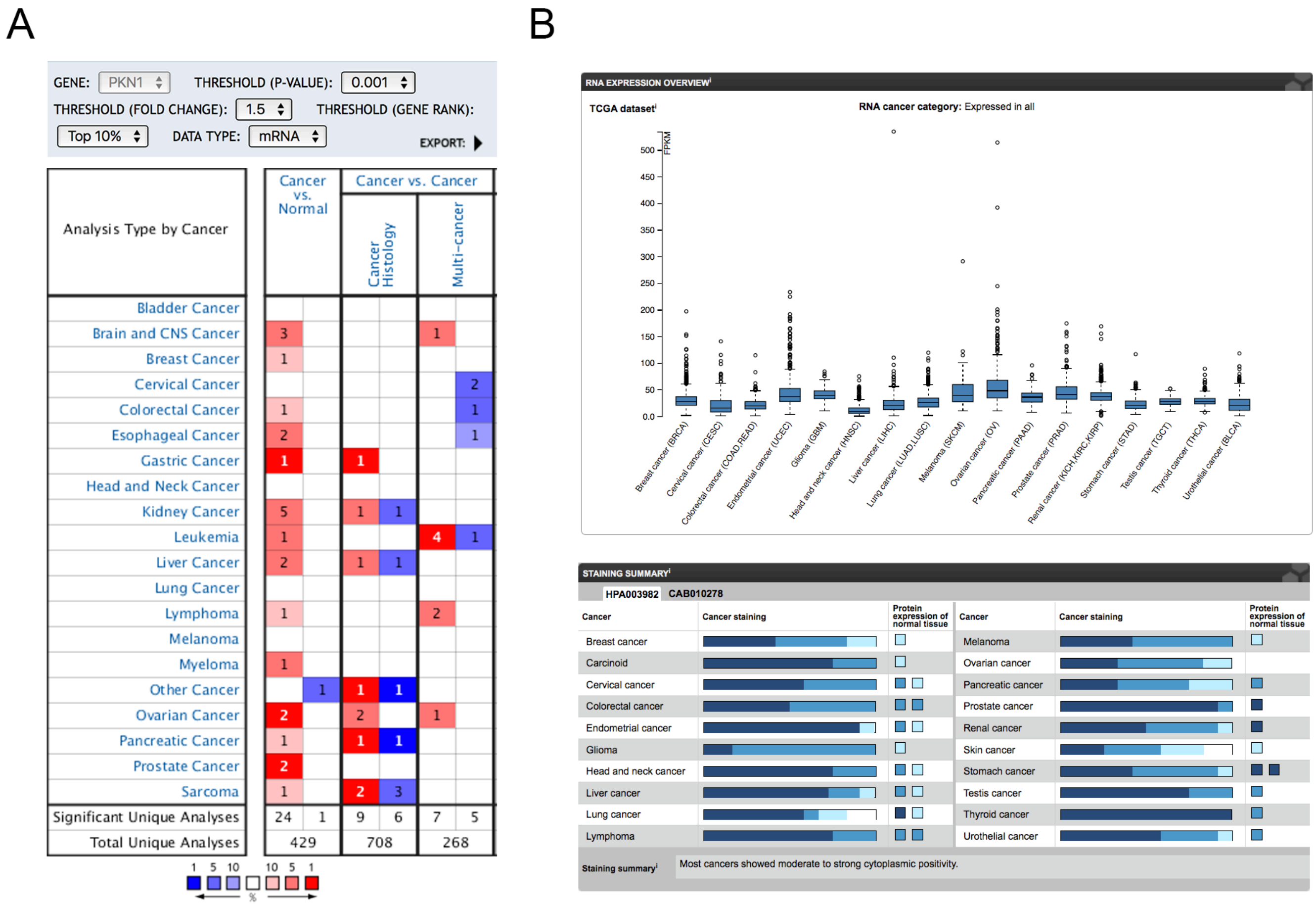

Supplementary Figure 7. PKN1 is upregulated in multiple cancers. (A) Data was obtained from the Oncomine database (https://www.oncomine.org/) with a threshold being *P* values < 0.001, fold change > 1.5 and gene ranks in top 10%. **(B)** Data was obtained from the Human Protein Atlas database (https://www.proteinatlas.org/).

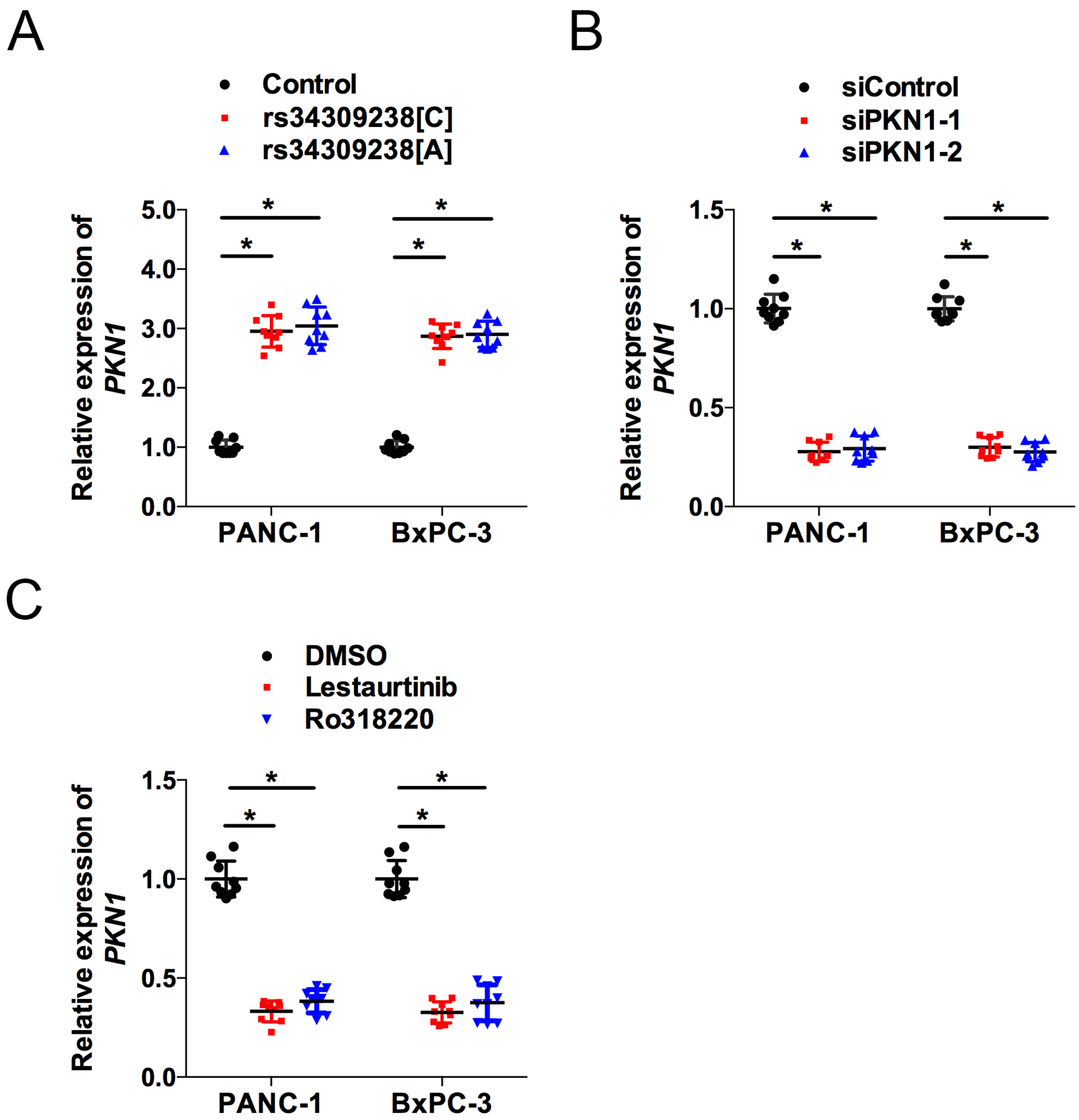

Supplementary Figure 8. Result of the test of the transfection efficiency. Relative expression levels of *PKN1* as determined by qRT–PCR in PANC-1 and BxPC-3 cells transfected with PKN1[C], PKN1[A] and control vector (**A**), or targeting siRNAs and siControl (**B**), or PKN1 inhibitors and DMSO as control (**C**). Results present mean ± SD from three independent experiments and each had three technical replications. *$P < 0.01$, compared with control cells by two-sided unpaired Student's *t*-test.

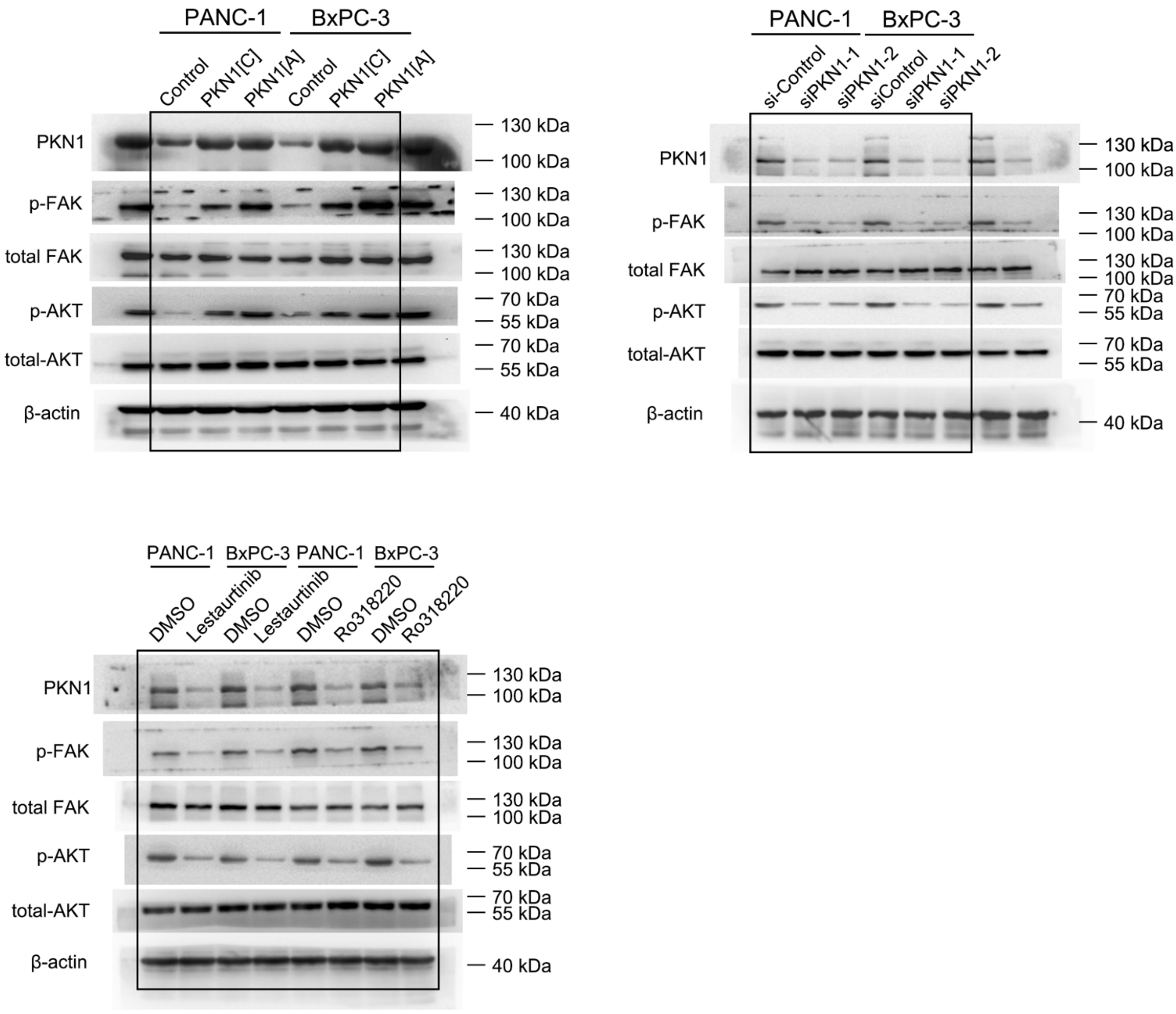

Supplementary Figure 9. Full scan of the western blot plots. The full scan of the western blot plots presented in Fig. 2C and D.
